# *N*-(4-Hydroxy­phen­yl)benzene­sulfon­amide

**DOI:** 10.1107/S160053681001322X

**Published:** 2010-04-14

**Authors:** Islam Ullah Khan, Irfana Mariam, Muhammad Zia-ur-Rehman, Muhammad Arif Sajjad, Shahzad Sharif

**Affiliations:** a Materials Chemistry Laboratory, Department of Chemistry, Government College University, Lahore 54000, Pakistan; b Applied Chemistry Research Centre, PCSIR Laboratories Complex, Lahore 54600, Pakistan

## Abstract

The title compound, C_12_H_11_NO_3_S, synthesized by the reaction of benzene sulfonyl chloride with *para*-amino­phenol, is of inter­est as a precursor to biologically active sulfur-containing heterocyclic compounds. The structure is stabilized by N—H⋯O and O—H⋯O hydrogen bonds.

## Related literature

For the synthesis of related mol­ecules, see: Zia-ur-Rehman *et al.* (2006 ▶, 2009 ▶). For a related structure, see: Khan *et al.* (2009 ▶).
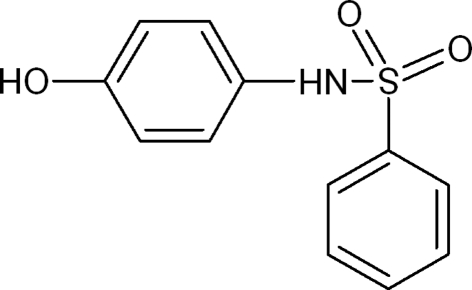


## Experimental

### 

#### Crystal data

C_12_H_11_NO_3_S
*M* *_r_* = 249.28
Orthorhombic, 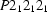

*a* = 5.1072 (2) Å
*b* = 9.3948 (4) Å
*c* = 24.0903 (10) Å
*V* = 1155.88 (8) Å^3^
*Z* = 4
Mo *K*α radiation
μ = 0.28 mm^−1^
*T* = 296 K
0.12 × 0.12 × 0.10 mm

#### Data collection

Bruker APEXII CCD area-detector diffractometer
6402 measured reflections
2808 independent reflections
2076 reflections with *I* > 2σ(*I*)
*R*_int_ = 0.032

#### Refinement

*R*[*F* ^2^ > 2σ(*F* ^2^)] = 0.048
*wR*(*F* ^2^) = 0.146
*S* = 1.02
2808 reflections
155 parameters
H-atom parameters constrained
Δρ_max_ = 0.26 e Å^−3^
Δρ_min_ = −0.26 e Å^−3^
Absolute structure: Flack (1983 ▶), with 1118 Friedel pairs
Flack parameter: 0.08 (13)

### 

Data collection: *APEX2* (Bruker, 2007 ▶); cell refinement: *SAINT* (Bruker, 2007 ▶); data reduction: *SAINT*; program(s) used to solve structure: *SHELXS97* (Sheldrick, 2008 ▶); program(s) used to refine structure: *SHELXL97* (Sheldrick, 2008 ▶); molecular graphics: *PLATON* (Spek, 2009 ▶) and *Mercury* (Macrae *et al.*, 2006 ▶); software used to prepare material for publication: *PLATON*.

## Supplementary Material

Crystal structure: contains datablocks I, global. DOI: 10.1107/S160053681001322X/bt5239sup1.cif

Structure factors: contains datablocks I. DOI: 10.1107/S160053681001322X/bt5239Isup2.hkl

Additional supplementary materials:  crystallographic information; 3D view; checkCIF report

## Figures and Tables

**Table 1:** Hydrogen-bond geometry (Å, °)

*D*—H⋯*A*	*D*—H	H⋯*A*	*D*⋯*A*	*D*—H⋯*A*
N1—H1N⋯O2^i^	0.96	2.07	3.030 (3)	173
O3—H3⋯O1^ii^	0.82	2.05	2.857 (4)	166

Symmetry codes: (i) 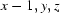; (ii) 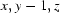.

**Fig. 1.**
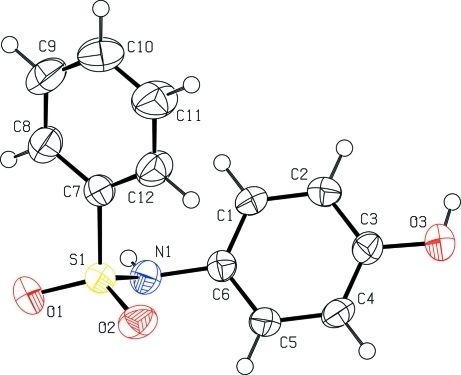
The molecular structure of the title compound with displacement ellipsoids at the 50% probability level.

**Fig. 2.**
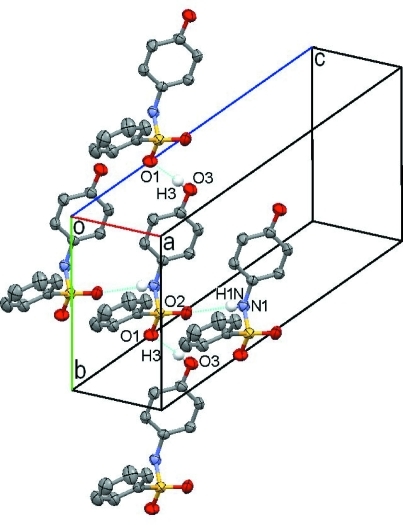
Perspective view of the three-dimensional crystal packing showing hydrogen-bonded interactions (dashed lines). H atoms not involved in hydrogen bonding have been omitted for clarity.

## supplementary crystallographic information

### Comment

In the present paper, the structure of *N*-(4-hydroxyphenyl) benzene sulfonamide (Fig. 1) has been determined as part of a research program involving the synthesis and biological evaluation of sulfur containing heterocyclic compounds (Zia-ur-Rehman *et al.*, 2006, 2009; Khan *et al.*, 2009). Bond lengths and bond angles are almost similar to those in the related molecules (Khan *et al.*, 2009). The molecules are linked through intermolecular N—H···O and O—H···O hydrogen bonds (Fig. 2; Table 1).

### Experimental

A mixture of benzene sulfonyl chloride (10.0 mmoles; 1.766 g), para-aminophenol (10.0 mmoles; 1.286 g), aqueous sodium carbonate (10%; 10.0 ml) and water (25 ml) was stirred for half an hour at room temperature followed by evaporation of the solvent. The crude mixture was washed with water and dried. Product obtained was dissolved in methanol and crystallized by slow evaporation of the solvent. Yield 73%.

### Refinement

All H atoms were identified in the difference map. Nevertheless, they were fixed in ideal positions and treated as riding on their parent atoms. The following distances were used: C_methyl_—H 0.98 Å, C_aromatic_—H 0.95Å, O—H 0.84 Å. U(H) was set to 1.2U_eq_ of the parent atoms or 1.5U_eq_ for methyl groups.

### Crystal data

**Table:**

C_12_H_11_NO_3_S	*F*(000) = 520
*M**_r_* = 249.28	*D*_x_ = 1.432 Mg m^−^^3^
Orthorhombic, *P*2_1_2_1_2_1_	Mo *K*α radiation, λ = 0.71073 Å
Hall symbol: P 2ac 2ab	Cell parameters from 6462 reflections
*a* = 5.1072 (2) Å	θ = 2.5–27.1°
*b* = 9.3948 (4) Å	µ = 0.28 mm^−^^1^
*c* = 24.0903 (10) Å	*T* = 296 K
*V* = 1155.88 (8) Å^3^	Needle, colourless
*Z* = 4	0.12 × 0.12 × 0.10 mm

### Data collection

**Table:**

Bruker APEXII CCD area-detector diffractometer	2076 reflections with *I* > 2σ(*I*)
Radiation source: fine-focus sealed tube	*R*_int_ = 0.032
graphite	θ_max_ = 28.3°, θ_min_ = 1.7°
φ and ω scans	*h* = −4→6
6402 measured reflections	*k* = −8→12
2808 independent reflections	*l* = −32→32

### Refinement

**Table:**

Refinement on *F*^2^	Secondary atom site location: difference Fourier map
Least-squares matrix: full	Hydrogen site location: inferred from neighbouring sites
*R*[*F*^2^ > 2σ(*F*^2^)] = 0.048	H-atom parameters constrained
*wR*(*F*^2^) = 0.146	*w* = 1/[σ^2^(*F*_o_^2^) + (0.0829*P*)^2^] where *P* = (*F*_o_^2^ + 2*F*_c_^2^)/3
*S* = 1.02	(Δ/σ)_max_ < 0.001
2808 reflections	Δρ_max_ = 0.26 e Å^−^^3^
155 parameters	Δρ_min_ = −0.26 e Å^−^^3^
0 restraints	Absolute structure: Flack (1983), 1118 Friedel pairs
Primary atom site location: structure-invariant direct methods	Flack parameter: 0.08 (13)

### Special details

**Table:**

Geometry. All esds (except the esd in the dihedral angle between two l.s. planes) are estimated using the full covariance matrix. The cell esds are taken into account individually in the estimation of esds in distances, angles and torsion angles; correlations between esds in cell parameters are only used when they are defined by crystal symmetry. An approximate (isotropic) treatment of cell esds is used for estimating esds involving l.s. planes.
Refinement. Refinement of *F*^2^ against ALL reflections. The weighted *R*-factor wR and goodness of fit *S* are based on *F*^2^, conventional *R*-factors *R* are based on *F*, with *F* set to zero for negative *F*^2^. The threshold expression of *F*^2^ > σ(*F*^2^) is used only for calculating *R*-factors(gt) etc. and is not relevant to the choice of reflections for refinement. *R*-factors based on *F*^2^ are statistically about twice as large as those based on *F*, and *R*- factors based on ALL data will be even larger.

### Fractional atomic coordinates and isotropic or equivalent isotropic displacement parameters (Å^2^)

**Table:**

	*x*	*y*	*z*	*U*_iso_*/*U*_eq_	
S1	0.63260 (16)	0.61227 (9)	0.12889 (3)	0.0363 (2)	
O1	0.5233 (5)	0.7519 (3)	0.13349 (12)	0.0522 (7)	
N1	0.4626 (5)	0.5129 (3)	0.17054 (11)	0.0370 (7)	
C1	0.4079 (7)	0.2576 (4)	0.15217 (13)	0.0395 (8)	
H1	0.2692	0.2783	0.1285	0.047*	
O2	0.9034 (4)	0.5892 (3)	0.14085 (9)	0.0485 (6)	
C2	0.4814 (6)	0.1180 (4)	0.16065 (13)	0.0431 (8)	
H2	0.3893	0.0445	0.1437	0.052*	
O3	0.7825 (6)	−0.0468 (3)	0.20338 (12)	0.0585 (8)	
H3	0.6871	−0.1039	0.1874	0.088*	
C3	0.6923 (7)	0.0881 (4)	0.19449 (13)	0.0396 (8)	
C4	0.8231 (7)	0.1962 (4)	0.22069 (14)	0.0433 (9)	
H4	0.9647	0.1759	0.2437	0.052*	
C5	0.7442 (7)	0.3353 (4)	0.21288 (14)	0.0402 (8)	
H5	0.8313	0.4087	0.2311	0.048*	
C6	0.5380 (6)	0.3661 (4)	0.17841 (12)	0.0321 (7)	
C7	0.5766 (6)	0.5525 (4)	0.06026 (12)	0.0351 (7)	
C8	0.3782 (7)	0.6111 (4)	0.02947 (14)	0.0493 (8)	
H8	0.2742	0.6831	0.0442	0.059*	
C9	0.3350 (8)	0.5617 (5)	−0.02374 (15)	0.0595 (11)	
H9	0.2018	0.6009	−0.0452	0.071*	
C10	0.4879 (8)	0.4549 (5)	−0.04506 (15)	0.0598 (11)	
H10	0.4584	0.4225	−0.0810	0.072*	
C11	0.6818 (8)	0.3963 (5)	−0.01419 (16)	0.0662 (12)	
H11	0.7834	0.3233	−0.0289	0.079*	
C12	0.7288 (7)	0.4448 (4)	0.03900 (14)	0.0507 (9)	
H12	0.8620	0.4049	0.0602	0.061*	
H1N	0.2803	0.5303	0.1630	0.061*	

### Atomic displacement parameters (Å^2^)

**Table:**

	*U*^11^	*U*^22^	*U*^33^	*U*^12^	*U*^13^	*U*^23^
S1	0.0375 (4)	0.0329 (4)	0.0385 (4)	−0.0016 (4)	−0.0041 (3)	−0.0002 (4)
O1	0.0636 (17)	0.0303 (12)	0.0625 (16)	0.0040 (12)	−0.0060 (14)	−0.0046 (12)
N1	0.0364 (15)	0.0373 (15)	0.0373 (13)	0.0033 (12)	0.0024 (11)	0.0021 (13)
C1	0.0377 (18)	0.0422 (19)	0.0385 (16)	0.0000 (16)	−0.0116 (14)	0.0006 (15)
O2	0.0358 (13)	0.0577 (16)	0.0519 (13)	−0.0093 (12)	−0.0081 (11)	0.0039 (12)
C2	0.0430 (18)	0.0401 (18)	0.0463 (17)	−0.0019 (17)	−0.0108 (14)	−0.0059 (18)
O3	0.072 (2)	0.0341 (14)	0.0689 (18)	0.0048 (14)	−0.0216 (14)	0.0013 (14)
C3	0.048 (2)	0.0371 (19)	0.0340 (16)	0.0018 (16)	−0.0018 (13)	0.0043 (15)
C4	0.046 (2)	0.045 (2)	0.0395 (17)	−0.0033 (17)	−0.0152 (16)	0.0063 (16)
C5	0.0486 (19)	0.0345 (18)	0.0375 (16)	−0.0059 (17)	−0.0104 (15)	0.0008 (15)
C6	0.0352 (15)	0.0326 (17)	0.0284 (13)	0.0008 (14)	0.0042 (12)	0.0024 (13)
C7	0.0345 (17)	0.0343 (17)	0.0364 (15)	−0.0052 (14)	0.0005 (13)	0.0036 (14)
C8	0.0475 (19)	0.050 (2)	0.0504 (19)	0.004 (2)	−0.0062 (17)	0.0066 (18)
C9	0.060 (3)	0.073 (3)	0.046 (2)	0.000 (2)	−0.0144 (19)	0.012 (2)
C10	0.065 (3)	0.078 (3)	0.0364 (18)	−0.003 (2)	0.0001 (18)	−0.009 (2)
C11	0.065 (3)	0.084 (3)	0.050 (2)	0.017 (3)	−0.0014 (18)	−0.017 (2)
C12	0.045 (2)	0.061 (2)	0.0468 (19)	0.012 (2)	−0.0053 (16)	−0.0020 (19)

### Geometric parameters (Å, °)

**Table:**

S1—O2	1.429 (2)	C4—H4	0.9300
S1—O1	1.430 (3)	C5—C6	1.372 (4)
S1—N1	1.622 (3)	C5—H5	0.9300
S1—C7	1.769 (3)	C7—C8	1.371 (5)
N1—C6	1.445 (4)	C7—C12	1.375 (5)
N1—H1N	0.9629	C8—C9	1.381 (5)
C1—C6	1.371 (5)	C8—H8	0.9300
C1—C2	1.380 (5)	C9—C10	1.371 (6)
C1—H1	0.9300	C9—H9	0.9300
C2—C3	1.380 (4)	C10—C11	1.355 (6)
C2—H2	0.9300	C10—H10	0.9300
O3—C3	1.365 (4)	C11—C12	1.381 (5)
O3—H3	0.8200	C11—H11	0.9300
C3—C4	1.370 (5)	C12—H12	0.9300
C4—C5	1.381 (5)		
			
O2—S1—O1	120.06 (15)	C6—C5—H5	119.8
O2—S1—N1	107.82 (15)	C4—C5—H5	119.8
O1—S1—N1	105.73 (15)	C1—C6—C5	119.7 (3)
O2—S1—C7	107.28 (15)	C1—C6—N1	121.3 (3)
O1—S1—C7	107.48 (16)	C5—C6—N1	119.0 (3)
N1—S1—C7	107.99 (15)	C8—C7—C12	120.8 (3)
C6—N1—S1	119.2 (2)	C8—C7—S1	119.9 (3)
C6—N1—H1N	116.4	C12—C7—S1	119.3 (3)
S1—N1—H1N	107.6	C7—C8—C9	119.0 (4)
C6—C1—C2	120.4 (3)	C7—C8—H8	120.5
C6—C1—H1	119.8	C9—C8—H8	120.5
C2—C1—H1	119.8	C10—C9—C8	120.2 (4)
C1—C2—C3	119.6 (3)	C10—C9—H9	119.9
C1—C2—H2	120.2	C8—C9—H9	119.9
C3—C2—H2	120.2	C11—C10—C9	120.5 (4)
C3—O3—H3	109.5	C11—C10—H10	119.7
O3—C3—C4	116.8 (3)	C9—C10—H10	119.7
O3—C3—C2	123.1 (3)	C10—C11—C12	120.2 (4)
C4—C3—C2	120.1 (3)	C10—C11—H11	119.9
C3—C4—C5	119.8 (3)	C12—C11—H11	119.9
C3—C4—H4	120.1	C7—C12—C11	119.3 (3)
C5—C4—H4	120.1	C7—C12—H12	120.3
C6—C5—C4	120.4 (3)	C11—C12—H12	120.3
			
O2—S1—N1—C6	−45.9 (3)	O2—S1—C7—C8	−152.9 (3)
O1—S1—N1—C6	−175.5 (2)	O1—S1—C7—C8	−22.5 (3)
C7—S1—N1—C6	69.7 (3)	N1—S1—C7—C8	91.1 (3)
C6—C1—C2—C3	−2.0 (5)	O2—S1—C7—C12	28.9 (3)
C1—C2—C3—O3	−177.7 (3)	O1—S1—C7—C12	159.3 (3)
C1—C2—C3—C4	1.8 (5)	N1—S1—C7—C12	−87.1 (3)
O3—C3—C4—C5	179.1 (3)	C12—C7—C8—C9	−0.9 (6)
C2—C3—C4—C5	−0.4 (5)	S1—C7—C8—C9	−179.1 (3)
C3—C4—C5—C6	−0.9 (5)	C7—C8—C9—C10	0.4 (6)
C2—C1—C6—C5	0.7 (5)	C8—C9—C10—C11	0.3 (6)
C2—C1—C6—N1	−179.4 (3)	C9—C10—C11—C12	−0.6 (7)
C4—C5—C6—C1	0.8 (5)	C8—C7—C12—C11	0.6 (5)
C4—C5—C6—N1	−179.2 (3)	S1—C7—C12—C11	178.8 (3)
S1—N1—C6—C1	−101.5 (3)	C10—C11—C12—C7	0.2 (6)
S1—N1—C6—C5	78.5 (3)		

### Hydrogen-bond geometry (Å, °)

**Table:**

*D*—H···*A*	*D*—H	H···*A*	*D*···*A*	*D*—H···*A*
N1—H1N···O2^i^	0.96	2.07	3.030 (3)	173
O3—H3···O1^ii^	0.82	2.05	2.857 (4)	166

Symmetry codes: (i) *x*−1, *y*, *z*; (ii) *x*, *y*−1, *z*.
